# The antiepileptic effect of *Centella asiatica* on the activities of Na^+^/K^+^, Mg^2+^ and Ca^2+^-ATPases in rat brain during pentylenetetrazol–induced epilepsy

**DOI:** 10.4103/0253-7613.64504

**Published:** 2010-04

**Authors:** Visweswari G., Siva Prasad K., Lokanatha V., W. Rajendra

**Affiliations:** Department of Zoology, Division of Molecular Biology, Sri Venkateswara University, Tirupati, Andhra Pradesh, India; 1 Department of Biotechnology, Dravidian University, Kuppam, Andhra Pradesh, India

**Keywords:** Anticonvulsant effect, ATPases, *Centella asiatica*, epilepsy, pentylenetetrazol

## Abstract

**Background:**

To study the anticonvulsant effect of different extracts of *Centella asiatica* (CA) in male albino rats with reference to Na^+^/K^+^, Mg^2+^ and Ca^2+^-ATPase activities.

**Materials and Methods:**

Male Wistar rats (150±25 g b.w.) were divided into seven groups of six each i.e. (a) control rats treated with saline, (b) pentylenetetrazol (PTZ)-induced epileptic group (60 mg/kg, i.p.), (c) epileptic group pretreated with n-hexane extract (n-HE), (d) epileptic group pretreated with chloroform extract (CE), (e) epileptic group pretreated with ethyl acetate extract (EAE), (f) epileptic group pretreated with n-butanol extract (n-BE), and (g) epileptic group pretreated with aqueous extract (AE).

**Results:**

The activities of three ATPases were decreased in different regions of brain during PTZ-induced epilepsy and were increased in epileptic rats pretreated with different extracts of CA except AE.

**Conclusion:**

The extracts of *C. asiatica*, except AE, possess anticonvulsant and neuroprotective activity and thus can be used for effective management in treatment of epileptic seizures.

## Introduction

Epilepsy, a common chronic nervous system disorder with repeated seizures affects 1-2% population worldwide.[1] There are likely to be multiple underlying cellular and molecular mechanisms responsible for various epileptiform phenomena, such as (a) imbalance between excitatory and inhibitory neurotransmission, (b) alterations in neurotransmitter expression and function, (c) channelopathies, and (d) aberrant neuronal synchronization. Purine nucleotides, in addition to serving as precursors of nucleic acids, play a critical role in diverse physiological functions such as energy metabolism, protein synthesis, regulation of enzyme activity, and signal transduction.[2]

ATPases play an important role in the maintenance of ionic gradient by coupling ATP hydrolysis with energy processes.[3] ATP itself, as a neurotransmitter and neuromodulator, may influence the release of other neurotransmitters by acting through its own receptors or by altering the neurotransmitter receptors.[4,5] Na^+^, K^+^ ATPase is a membrane-bound enzyme and inactivation of this enzyme is an important factor in epileptization of neurons.[6] Similarly it is demonstrated that inhibition of microsomal Mg^2+^/Ca^2+^-ATPase may be associated with long-term plasticity changes associated with epileptogenesis.[7] Idiopathic epilepsies involve the Na^+^, K^+^, or Ca^2+^ channels and the defects in the activities of Na^+^/K^+^ or Ca^2+^-ATPases are responsible for maintaining ionic balance in the cell.[8] Several studies have reported significant inhibition of ATPase activity in the brain during different types of epileptic seizures.[9‐11] Studies have also shown alterations in the activities of ATPases in different models of epilepsy and modulation of ATP hydrolysis by antiepileptic drug treatment.[12,13] Although the prognosis for seizure control is good in at least 60% patients, upto 40% individuals suffer from intractable, pharmacoresistant epilepsy.[14,15] Recently, focus on ethnopharmacology research has increased all over the world and a growing body of evidence has indicated immense potential of medicinal plants as alternative and complementary therapies for many human ailments.

Gotu kola, scientifically named as *Centella asiatica*, of the Apiaceae (Umbelliferae) family is an herbaceous annual plant native to the Asia. The most important bioactive components of *C. asiatica* leaf are triterpenoid glycosides (asiatic acid, medacassic acid), saponin glycosides (Brahmiside, Brahminoside), and flavonoids.[16,17] Gotu kola extract was reported to be beneficial in improving memory and also for the treatment of mental fatigue and anxiety.[18,19] CA reverses the effect of a GABA_A_ antagonist, PTZ, and protects against PTZ-induced convulsions and ATPase inhibition.[20,21] Asiaticoside, an active constituent in methanol and ethylacetate extracts of *C. asiatica*, has anxiolytic activity.[22] Keeping in view of neuroprotective role of this medicinal plant, the present study has been taken up to examine the alterations in the activities of all three ATPases in different regions of brain during PTZ-induced epilepsy and on antiepliptic treatment with CA extracts.

## Materials and Methods

### Collection of Plant Material

The leaves of *C. asiatica* used in this work were collected from Tirumala Hills, Andhra Pradesh, India, and authenticated by qualified botanist at Department of Botany, Sri Venkateswara University, Tirupati, Andhra Pradesh, India. The leaves were shade-dried and reduced to coarse powder.

### Extract Preparation

The extraction was carried out as specified by Sowmyalakshmi[23] and Vattanajun.[24] The powdered plant material was soaked in methanol for 2 days at room temperature and the solvent was filtered. This was repeated three to four times until the extract gave no coloration. It was distilled and concentrated under reduced pressure in the Buchi rotavapour R-114 yielding a gum-like residue, which was then suspended in water and extracted with various organic solvents of increasing polarity (starting with the lipophilic solvent n-Hexane, ending with the more hydrophilic n-Butanol). The solvent from each extract was distilled and concentrated under reduced pressure in the Buchi rotavapor. Finally, the extracts were freeze dried and used for further studies.

### Experimental Animals

Adult male Wistar rats (150±25g) were used for the present study. The experimental rats were housed in polypropylene cages under laboratory conditions of 28±2°C temperature with 75% relative humidity and photoperiod of 12 h light/dark cycle. The rats were given standard pellet diet (Gold Mohur Food and Feeds Ltd., Bangalore) and water *ad libitum*. The rats were maintained according to the ethical guidelines for animal protection and welfare bearing the CPCSEA 438/01/a/cpcsea/dt 17.07.2006 in its resolution No: 9/IAEC/SVU/2006/dt 04.03.2006.

### Induction of Epilepsy

Convulsions were induced by an injection of pentylenetetrazol (60 mg/kg b.w., i.p.) in saline.[25]

### Administration of Test Substance

Each fraction of CA extract (200 mg/kg b.w.) was dissolved in saline and given to the animals for 1 week prior to the injection of PTZ.[26] A gavage tube was used to deliver the substance by the oral route, which is the clinically expected route of administration of *C. asiatica*. The volume of administration was kept at 1 ml/kg b.w.

### Drugs and Chemicals

Pentylenetetrazol was obtained from Sigma-Aldrich, St. Louis, MO, USA. All other chemicals were of analytical grade.

### Isolation of Tissues

After stipulated duration, the animals were sacrificed by cervical dislocation and different brain regions such as cerebral cortex (CC), cerebellum (CB), pons medulla (PM), and hippocampus (HC) were immediately isolated, frozen in liquid nitrogen, and stored at -80°C until analysis.

### Biochemical Analysis

#### ATPases (E.C. 3.6.1.3):(ATP Phosphohydrolase)

The activities of ATPases were assayed by the method of Fritz and Hamrick[27] as reported by Desaiah and Ho[28] with slight modifications and the inorganic phosphate liberated was estimated by the method of Lowry and Lopez[29] as modified by Phillips and Hayes.[30]

Tissue homogenates were prepared in ice cold 0.32 M sucrose containing 1.0 mM EDTA and 10 mM imidazole (pH 7.5). The homogenates were centrifuged at 1000 × *g* and the supernatant obtained was used as an enzyme source.

#### Na^+^, K^+^-ATPase

The reaction mixture in a total volume of 3.0 ml contained 3 mM ATP, 3 mM MgCl_2_, 100 mM NaCl, 20 mM KCl, 135 mM imidazole-HCl buffer (pH 7.5) and 10 mg of tissue. The reaction mixture was incubated at 37°C for 30 min and stopped by the addition of 0.1 ml of 50% TCA. Samples were then assayed for inorganic phosphate by the method of Lowry and Lopez[29] as modified by Phillips and Hayes.[30] The color intensity was read at 800 nm in a spectrophotometer (Hitachi). Mg^2+^ - ATPase activity was measured in the presence of 1 mM Ouabain, a specific inhibitor of Na^+^, K^+^ - ATPase. Ouabain-sensitive Na^2+^, K^+^-ATPase activity was obtained by the difference between total ATPase and Mg^2+^-ATPase activity. The enzyme activity was expressed as *µ*moles of inorganic phosphate formed/mg protein/hr.

#### Ca^2+^-ATPase activity

The reaction mixture in a total volume of 3 ml contained 135 mM imidazole – HCl buffer (pH 7.5), 5 mM MgCl_2_, 0.05 mM CaCl_2_, 4 mM ATP, and 30 mg of tissue. The mixture was incubated at 37°C for 30 min and stopped by the addition of 0.1 ml of 50% TCA. The inorganic phosphate formed was estimated by the method of Lowry and Lopez[29] as modified by Phillips and Hayes.[30] Mg^2+^ - ATPase activity was measured in the presence of 0.5 mM EGTA and this value was subtracted from total ATPase activity to get Ca^2+^-ATPase activity. Enzyme activity was expressed as *µ*moles of inorganic phosphate formed/mg protein/h.

### Statistical Analyses

Mean, standard deviation (SD), and analyses of variance (ANOVA) for the data were calculated by using the SPSS (Statistical Package for Social Sciences) software.

## Results

The activity levels of Na^+^, K^+^ -ATPase, Mg^2+^-ATPase, and Ca^2+^-ATPase were studied in different regions of brain during PTZ-induced epilepsy and on pre-treatment with different extracts of *C. asiatica*. In general, all the three ATPases showed conspicuous inhibition during PTZ-induced epilepsy in all the brain regions (CC, CB, PM, and HC) when compared to respective saline controls. On pre-treatment with different CA extracts, the activity levels, in general, were increased in all the brain regions except for the treatment with aqueous extract (AE) when compared to the respective PTZ-induced epileptic group [Tables 1‐3].

**Table 1:** Changes in the sodium, potassium-ATPase (Na^+^/K^+^ -ATPase) in different regions of rat brain during PTZ-induced epilepsy and on pretreatment with different extracts of *Centella asiatica*

*Brain area*	*SC*	*PTZ*	*nHE+PTZ*	*CE+PTZ*	*EAE+PTZ*	*nBE+PTZ*	*AE+PTZ*	*F-value*
CC	4.491	3.909*	6.206*	4.564	4.796	7.074*	3.405*	83.75
	±0.300	±0.467	±0.282	±0.287	±0.196	±0.546	±0.601	
		(-12.96)	(38.18)	(1.62)	(6.78)	(57.50)	(-24.19)	
CB	4.929	3.876*	5.779	5.141	5.135	7.117*	3.502*	43.46
	±0.189	±0.426	±0.444	±0.495	±0.570	±0.597	±0.132	
		(-21.36)	(17.24)	(4.31)	(4.19)	(44.38)	(-28.94)	
PM	5.64	4.138*	8.054*	4.803	5.741	7.847*	3.609*	76.36
	±0.311	±0.443	±0.449	±0.189	±0.582	±0.524	±0.377	
		(-26.64)	(42.79)	(-14.83)	(1.77)	(39.12)	(-36.00)	
HC	5.273	4.384	6.729*	6.206*	8.736*	4.679	3.933*	82.73
	±0.609	±0.261	±0.234	±0.282	±0.228	±0.277	±0.519	
		(-16.85)	(27.62)	(17.70)	(65.68)	(-11.26)	(-25.41)	

Values are expressed in μ moles of inorganic phosphate formed/mg protein/h., All the values are mean, ±SD of five individual observations, Values in '()' parentheses are percent change over saline control, Values with

* are significant at *P*<0.05 compared to SC in Scheffe test

**Table 2:** Changes in the sodium, potassium-ATPase (Na^+^/K^+^ -ATPase) in different regions of rat brain during PTZ-induced epilepsy and on pretreatment with different extracts of *Centella asiatica*

*Brain area*	*SC*	*PTZ*	*nHE+PTZ*	*CE+PTZ*	*EAE+PTZ*	*nBE+PTZ*	*AE+PTZ*	*F-value*
CC	7.771	5.039*	6.152*	6.210*	6.531*	7.719	4.646*	23.41
	±0.220	±0.512	±0.562	±0.463	±0.483	±0.504	±0.341	
		(-35.14)	(-20.82)	(-20.08)	(-15.95)	(-0.65)	(-40.2)	
CB	6.935	5.547*	9.469*	6.769	6.544	5.993*	4.646*	123.17
	±0.410	±0.375	±0.211	±0.222	±0.439	±0.199	±0.341	
		(-20.01)	(36.55)	(-2.39)	(-5.62)	(-13.58)	(-32.99)	
PM	5.122	3.652*	7.501*	5.602	6.673*	4.542	3.255*	110.99
	±0.687	±0.335	±0.280	±0.382	±0.315	±0.304	±0.275	
		(-28.69)	(46.46)	(9.38)	(30.28)	(-11.31)	(-36.43)	
HC	6.708	4.689*	5.150*	5.626*	8.673*	6.831	4.330*	78.65
	±0.252	±0.646	±0.215	±0.332	±0.282	±0.234	±0.521	
		(-30.09)	(-23.21)	(-16.12)	(29.30)	(1.83)	(-35.45)	

Values are expressed in μmoles of inorganic phosphate formed/mg protein/h., All the values are mean, ±SD of five individual observations, Values in '()' parentheses are percent change over saline control, Values with

* are significant at *P*<0.05 compared to SC in Scheffe test

**Table 3:** Changes in the calcium-ATPase (Ca^2^ ± -ATPase) in different regions of rat brain during PTZ-induced epilepsy and on pre-treatment with different extracts of *Centella asiatica*

*Brain area*	*SC*	*PTZ*	*nHE+PTZ*	*CE+PTZ*	*EAE+PTZ*	*nBE+PTZ*	*AE+PTZ*	*F-value*
CC	5.844	4.31*	5.593	7.543*	7.417*	6.231	4.126*	40.06
	±0.675	±0.573	±0.392	±0.441	±0.565	±0.258	±0.698	
		(-26.24)	(-4.29)	(29.06)	(26.91)	(6.62)	(-29.40)	
CB	5.31	3.898*	5.714	4.970	6.953*	6.192	3.164*	58.88
	±0.940	±0.663	±0.681	±0.221	±0.478	±0.296	±0.243	
		(-26.59)	(7.61)	(-6.40)	(30.93)	(16.61)	(-40.41)	
PM	5.089	3.493*	4.234	6.566	6.030	5.456	3.110*	16.36
	±0.731	±0.298	±0.511	±1.177	±0.753	±0.635	±0.418	
		(-31.34)	(-16.79)	(29.02)	(18.49)	(7.22)	(-38.87)	
HC	6.072	3.284*	8.033*	4.660*	6.674	5.044	3.146*	66.14
	±0.704	±0.204	±0.333	±0.294	±0.281	±0.687	±0.251	
		(-45.90)	(32.30)	(-23.25)	(9.92)	(-16.92)	(-48.18)	

Values are expressed in μ moles of inorganic phosphate formed/mg protein/h., All the values are mean, ±SD of five individual observations, Values in '()' parentheses are percent change over saline control, Values with

* are significant at *P*<0.05 compared to SC in Scheffe test

## Discussion

Mitochondrial dysfunction and consequent energy depletion are the major causes of oxidative stress and alterations in the ionic homeostasis in the central nervous system which causes loss of cellular integrity and cell death. Mitochondrial dysfunction, excitotoxicity, and generation of reactive oxygen species have been associated with various CNS disorders such as seizures, Parkinsonism, Huntington, and Alzheimer's diseases.[31,32]

In the present investigation, a decrease in Na^+^, K^+^-ATPase, Mg^2+^-ATPase, and Ca^2+^-ATPase activities was observed in all the brain areas during PTZ-induced epilepsy, which is in agreement with the earlier reports.[20,33] Inhibition of Na^+^, K^+^-ATPase in monkey and human epileptic brain and inhibition of Ca^2+^-ATPase in seizure prone mice have also been reported.[8] Similar inhibition of Na^+^, K^+^-ATPase activity was also recorded in hippocampus and cortex during kainic acid-induced epilepsy.[33] Since ATPases play a pivotal role in maintenance of cellular ionic gradient, their inhibition probably enhance neuronal excitability and facilitates the appearance of excitatory activity and convulsions.[34] Accordingly, ATPase inhibition leads to uncontrolled dendrite discharge in purkinje cells of rat cerebellum and causes electrographically recorded seizures in mice.[35] Since the Na^+^, K^+^ and Ca^2+^ ions are important in the development and conduction of action potential, the decrease in the activities of respective ATPases may alter the rate of influx and efflux of cations correlating with altered membrane permeability properties. Further, the decrement in the activities of ATPases reflects the decreased turnover of ATP, presumably due to inhibition of the oxidoreductase system and uncoupling of oxidative phosphorylation.

All the three ATPases were elevated in different regions of brain during pre-treatment with CA extracts in PTZ-induced epileptic animals except for the treatment with AE. Although not related to the present studies, it has been shown that *Curcumin* plays neuroprotective role by elevating Na^+^, K^+^-ATPase activity in all the brain regions of rat.[36] Studies with Bacoside A isolated from *Bacopa monniera* showed that administration of Bacoside A inhibited lipid peroxidation, improved the activities of Na^+^, K^+^-ATPase, Ca^2+^-ATPase, and Mg^2+^-ATPase, and maintained the ionic equilibrium.[37] The present findings coupled with earlier reports suggest that the bioactive factors present in selected extracts of CA offer neuroprotection by directly or indirectly modulating the activities of ATPases and thus may be helpful in the treatment of seizure disorders.
